# The *Arabidopsis LYST INTERACTING PROTEIN 5* Acts in Regulating Abscisic Acid Signaling and Drought Response

**DOI:** 10.3389/fpls.2016.00758

**Published:** 2016-06-01

**Authors:** Zongliang Xia, Yongjin Huo, Yangyang Wei, Qiansi Chen, Ziwei Xu, Wei Zhang

**Affiliations:** ^1^College of Life Science, Henan Agricultural UniversityZhengzhou, China; ^2^Zhengzhou Tobacco Research Institute of CNTCZhengzhou, China; ^3^China National Tobacco Quality Supervision and Test CentreZhengzhou, China

**Keywords:** *Arabidopsis*, abscisic acid, drought, MVB biogenesis

## Abstract

Multivesicular bodies (MVBs) are unique endosomes containing vesicles in the lumens and play essential roles in many eukaryotic cellular processes. The *Arabidopsis* LYST INTERACTING PROTEIN 5 (LIP5), a positive regulator of MVB biogenesis, has critical roles in biotic and abiotic stress responses. However, whether the abscisic acid (ABA) signaling is involved in LIP5-mediated stress response is largely unknown. Here, we report that *LIP5* functions in regulating ABA signaling and drought response in *Arabidopsis*. Analyses of a *LIP5* promoter-β-glucuronidase (*GUS*) construct revealed substantial GUS activity in whole seedlings. The expression of *LIP5* was induced by ABA and drought, and overexpression of *LIP5* led to ABA hypersensitivity, enhanced stomatal closure, reduced water loss, and, therefore, increased drought tolerance. On the contrary, *LIP5* knockdown mutants showed ABA-insensitive phenotypes and reduced drought tolerance; suggesting that *LIP5* acts in regulating ABA response. Further analysis using a fluorescent dye revealed that ABA and water stress induced cell endocytosis or vesicle trafficking in a largely LIP5-dependent manner. Furthermore, expression of several drought- or ABA-inducible marker genes was significantly down-regulated in the *lip5* mutant seedlings. Collectively, our data suggest that *LIP5* positively regulates drought tolerance through ABA-mediated cell signaling.

## Introduction

Adverse environmental conditions such as drought and high salinity have been becoming major limiting factors for plant growth and development. When encountering these stressful conditions, plants can respond and adapt to the adverse environment by triggering a network of signaling events to maintain cellular function (Hasegawa et al., 2000; Kawasaki et al., 2001; Zhu, 2002). Although, numerous drought-responsive genes have been characterized (Xiong et al., 2002; Zhu, 2002), the biological functions of many of these genes are essentially unknown in plants. Thus, it is desirable to understand the roles of these genes to improve crop adaptation to drought stress.

Abscisic acid (ABA) regulates many processes of plant growth and development, such as seed maturation, germination, and seedling growth (Leung and Giraudat, 1998; Finkelstein et al., 2002; Kermode, 2005; Christmann et al., 2006). Also, ABA is well-known for its regulatory roles in mediating adaptive responses to environmental stresses (Knight and Knight, 2001). For example, under drought stress, ABA induces stomatal closure to prevent water loss and launches a series of protective mechanisms to alleviate oxidative stress-induced cell damage in plants (Zhu, 2002; Yamaguchi-Shinozaki and Shinozaki, 2006).

In eukaryotic cells, multivesicular bodies (MVBs) are unique endosomes containing vesicles in the lumens. MVBs function as a protein degradation route in the endocytic pathway through which intraluminal vesicles can be delivered into and degraded upon fusion with lysosomes or vacuoles (Reyes et al., 2011; Contento and Bassham, 2012). In the MVB pathway, the ESCRT (endosomal sorting complexes required for transport) are dissociated or disassembled from the plasma membrane and recycled back into the cytoplasm (Reyes et al., 2011; Contento and Bassham, 2012). Hence, the MVB pathway has acted as a regulatory mechanism for removing damaged or down-regulated proteins from the plasma membrane. The Vps4p/SKD1 AAA ATPase together with its positive regulator Vta1/LIP5 catalyzes the process of ESCRT disassembly in an ATP-dependent reaction (Babst et al., 1998; Fujita et al., 2004; Scott et al., 2005; Azmi et al., 2006; Lottridge et al., 2006; Winter and Hauser, 2006). Studies in both yeast and mammalian cells have indicated that both Vps4p/SKD1 and Vta1/LIP5 are critical players during MVB biogenesis (Yeo et al., 2003; Shiflett et al., 2004; Ward et al., 2005; Azmi et al., 2006). In metazoans, the ESCRT-III protein CHMP5 functions as a negative allosteric switch to control LIP5-mediated stimulation of VPS4 (Vild et al., 2015).

In higher plants, *Arabidopsis* LIP5 interacts with the AAA ATPase SKD1 and increases its ATPase activity by 4–5 folds *in vitro* (Haas et al., 2007). SKD1 participates in MVB function and contributes to vacuolar maintenance (Shahriari et al., 2010). *Arabidopsis SKD1* knockout mutant is lethal and expression of the ATPase-deficient version SKD1^E232Q^ also causes alterations in the endosomal system and ultimately cell death (Haas et al., 2007). Surprisingly, loss of function of *LIP5* in *Arabidopsis* causes no phenotypic alterations under normal growth conditions, demonstrating that the activator LIP5 is dispensable for plant growth and development (Haas et al., 2007). Besides its function in MVB biogenesis, *Arabidopsis* LIP5 has been shown to play critical roles in biotic (resistance to *Pseudomonas syringae*; Wang et al., 2014) and abiotic stress tolerance (salt or heat stress; Wang et al., 2015). However, whether the ABA signaling is involved in LIP5-mediated stress response is largely unknown. In this study, we provide genetic evidence that *LIP5* functions in regulating ABA signaling and drought response in *Arabidopsis*.

## Materials and Methods

### Plant Materials and Growth Conditions

*Arabidopsis* (*Arabidopsis thaliana* ecotype Columbia [Col-0]) was used as the wild type in this study. Seeds of each genotype were surface sterilized with 10% bleach for 10 min and washed three times with sterile water. Sterilized seeds were then plated on Murashige and Skoog (MS) medium. Plants were stratified at 4°C in darkness for 3 days and then transferred to a growth chamber at 22°C with a 16-h-light/8-h-dark photoperiod. After 1 week, seedlings were potted in soil and placed in a growth room at 22°C, 60–70% relative humidity, a photoperiod of 16 h/8 h (day/night) and light intensity of 150 μmol m^-2^ s^-1^, as described previously (Xia et al., 2014).

### Verification of *LIP5 Arabidopsis* Mutants

The *LIP5* T-DNA insertion mutants SALK_123717 (named *lip5-s123*) and SALK_145666 (named *lip5-s145*) seeds were obtained from the ABRC collection center (Ohio State University, Columbus). Homozygous mutants were identified by PCR from genomic DNA using forward primer P1, T-DNA left border primer LBb1, and *LIP5* (accession number: At4g26750) gene-specific reverse primer P2 (Supplementary Table S1), and analyzed further by DNA sequencing to confirm the insertions of the T-DNA in the gene. The transcript levels of *LIP5* in the Col-0 and mutants were determined by quantitative RT-PCR.

### Real-Time PCR Analysis

Real-time PCR was used to determine the expression pattern of *LIP5* in different organs and in response to ABA treatment during seed germination and early seedlings growth. Total RNA extraction and first-strand cDNA synthesis were conducted as described previously (Xia et al., 2012). The qRT-PCR was performed in triplicate with an IQ5 light cycler system (Bio-Rad) using SYBR Premix ExTaq II (Takara, Japan) with gene-specific primers LIP5-QF and LIP5-QR (Supplementary Table S1), which produces a 157-bp product. The *Arabidopsis Actin2* transcript was used as an internal control to quantify the relative transcript levels as described by us previously (Xia et al., 2014). The relative levels of transcripts were detected using the 2^-ΔΔC^_T_ method (Livak and Schmittgen, 2001).

To examine the relative expression of *LIP5* in transgenic *Arabidopsis* plants, the expression of *Actin2* was used as an internal control, and the wild-type was regarded as a standard and the relative level of gene expression was computed as described above. All qRT-PCR experiments were performed with three biological and three technical replicates.

### Construction of Plant Expression Vectors and Development of Transgenic *Arabidopsis* Lines

For over-expression of *LIP5*, the *LIP5* coding sequence was amplified and introduced into the binary vector pMW101 using primers LIP5-F with *Bam*HI restriction site (underlined) and LIP5-R with *Xba*I restriction site (underlined; Supplementary Table S1), resulting in the transformation construct pMW101-35S:LIP5.

For *LIP5 promoter*:*GUS* fusion construct, a DNA fragment covering 1,281 bp upstream of the translational start site of *LIP5* was amplified by PCR using the primers LIP5P-F with *Eco*RI restriction site (underlined) and LIP5P-R with *Hin*dIII restriction site (underlined; Supplementary Table S1). This fragment was cloned into the *Eco*RI and *Hin*dIII sites of the binary vector pCAMBIA1381 containing a *GUS* reporter. GUS staining assay was done essentially as described previously (Bu et al., 2009).

The binary constructs were introduced into *Agrobacterium tumefaciens* (strain GV3101) and then transformed into *Arabidopsis* wild-type (Col-0) or *lip5* mutants via the floral dip method (Clough and Bent, 1998). Transgenic lines were selected by germinating seeds on MS medium containing 50 mg/L hygromycin. After 2 weeks on selection medium, green seedlings (T1 plants) were transferred to soil and grown to maturity to set seeds (T2 seeds) in a growth room. T2 seeds were germinated on antibiotics-selective medium again and the one-copy lines were identified by examining the segregation ratio (3:1) of the antibiotics-selectable marker. Each one-copy line was maintained growth to set seeds until T_3_ generation. Homozygous T3 lines were used for ABA response or GUS staining assays.

### *In silico* Analysis of *LIP5* Promoter

To identify putative *cis*-acting regulatory elements in the promoter region of *LIP5*, the online analysis tool PlantCARE^1^ was used.

### Root Growth of Seedlings

To study the effect of ABA on root growth of seedlings, seeds were sown on ABA-free medium as described above. 24 h after stratification, germinated seeds were transferred to medium containing different concentrations (0, 1, or 2 μM) of ABA. Plates were placed vertically in a growth chamber, and root growth was measured at 5 days after the end of stratification.

### Drought Treatment and Leaf Water Content Measurement

For gene expression analysis, 2-weeks-old seedlings from the agar plate were transferred onto a filter paper in a covered Petri dish and subjected to dehydration treatment. The treatment was conducted in an environment of 70% relative humidity. For the drought tolerance test on the soil-grown plants, 1-week-old seedlings were transplanted to the soil for additional 2 weeks under standard growth conditions, and then plants were subjected to progressive drought by withholding water for 12 days and then re-watered for 4 days. Survival rates (%) under drought treatment were determined as the number of visibly green plants after 4 days. To minimize experimental variations, the same number of plants was grown on the same tray. After 8 days without watering, weight of leaf samples from transgenic, mutant and wild-type plants under stressed and control conditions was determined as fresh weight (FW), and then the samples were dried at 80°C for 24 h and weighed as dry weight (DW). The water content was calculated as (FW–DW)/FW × 100%. The entire test was conducted at least three times.

### Stomatal Aperture Bioassay

Leaves from 3-weeks-old plants grown under the same conditions were harvested in darkness. Epidermal peels were stripped and incubated in a solution (50 mM KCl, 10 mM CaCl_2_, and 10 mM MES-KOH, pH 6.15), and exposed to light for 1.5 h. Subsequently, 50 μM ABA was added to the solution to assay for stomatal closing. After treatment for 3 h, stomatal apertures were measured as described (Xia et al., 2013) with a microscope. The apertures of usually 30–40 stomata were measured in two independent experiments.

### GUS Bioassays

Seeds and young seedlings at different developmental stages, and different parts from mature transgenic plants, were collected and used for histochemical detection of GUS expression (Jefferson et al., 1987). Materials were stained at 37°C overnight in the solution [1 mg/mL 5-bromo-4-chloro-3-indolyl-β-D-glucuronic acid (X-Gluc), 5 mM potassium ferricyanide, 5 mM potassium ferrocyanide, 0.03% Triton X-100, and 0.1 M sodium phosphate buffer, pH 7.0], and then were photographed after GUS staining.

### Fluorescence Microscopy

Fluorescent microscopy analysis of a styryl dye FM1-43 (Sigma-Aldrich) internalization in *Arabidopsis* root or leaf guard cells was conducted as described previously (Wang et al., 2015). For ABA-induced vesicle trafficking observation, the detached leaves from 2 weeks-old plants with 1 h-ABA (50 μM) treatment were used. For drought-induced vesicle trafficking observation, the excised roots from 10 days-old seedlings with 30 min-PEG 6000 (10%) treatment were collected. Leaf epidermal peels or root samples were stained in 10 μM of FM1-43 for 20 min at room temperature, washed and examined under a confocal microscope. For confocal laser microscope scanning, the wavelength of the confocal microscopy was set to be 488 nm excitation and 600–650 nm emission. About 8∼10 independent leaves or roots were quantified and statistically analyzed for each genotype/treatment.

### Accession Numbers

Sequence data from this article can be found in the GenBank/EMBL data libraries under the following accession numbers: *LIP5*, At4g26750; *RAB18*, At5g66400; *RD29A*, At5g52310; *RD29B*, At5g52300; *KIN1*, At5g15960; *RD22*, At5g25610; *ADH1*, At1g77120.

### Statistical Analysis

The data were presented as the mean ± standard error (SE) of three independent experiments. Statistical analyses were conducted using the data processing software SPSS V.16.0 (SPSS, Inc., USA). For all analyses, the significant level was set at *P* < 0.05.

## Results

### Expression Pattern of *LIP5*

**FIGURE 1:**
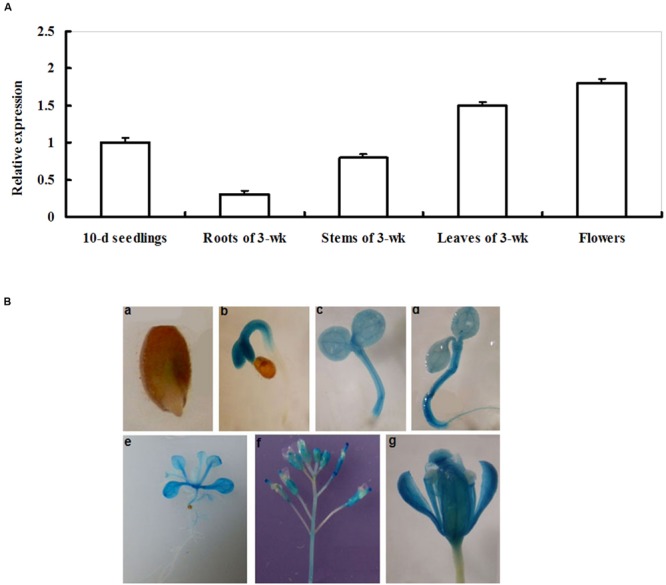
**Expression patterns of *LIP5.* (A)** qRT-PCR analysis of *LIP5* expression in different organs. Transcript levels of *LIP5* were quantified by qRT-PCR against *Actin2*. For each assay, the expression level at 10-days-old seedlings stage was defined as 1.0 and three technical replicates were conducted. Data shown are mean ± SE of three independent experiments. **(B)** GUS staining of *LIP5pro:GUS* transgenic plants from different growth stages. **(a)** 1-day-old germinating seedling; **(b)** 2-days-old seedling; **(c)** 3-days-old seedling; **(d)** 4-days-old seedling; **(e)** 2-weeks-old seedling; **(f)** inflorescences from 7-weeks-old plants; **(g)** flower from 7-weeks-old plants. Experiments were repeated at least two times with similar results.

The transcriptional pattern of *LIP5* was examined in young seedlings and in multiple organs of more mature plants by qRT-PCR. As shown in **Figure 1A**, the transcripts of *LIP5* were detected in young seedlings, roots, stems, leaves, and flowers of more mature plants. The *LIP5* transcript levels were significantly high in leaves and flowers. In contrast, *LIP5* transcripts were low in roots (**Figure 1A**).

For a more detailed analysis of the *LIP5* expression pattern, a 1,281-bp promoter sequence of *LIP5* was fused with the *GUS* gene to generate transgenic plants *LIP5pro:GUS* and to follow *LIP5* expression in different developmental stages. Histochemical staining showed GUS activity at all developmental stages tested, from seed germination to flowering (**Figure 1B**). This indicates that *LIP5* is expressed at all developmental stages and throughout the *Arabidopsis* plant. GUS expression was first detected in 1-day-old germinated seeds with weaker GUS staining in the emerging radicle (**Figure 1Ba**). Higher GUS expression was detected in hypocotyls of 2- and 3-days-old seedlings (**Figures 1Bb,c**). At 4 days, GUS expression was detected throughout the plant (**Figure 1Bd**). In 2- or 7-weeks-old plants, GUS staining was clearly observed in leaves or inflorescences, but relatively weak in roots (**Figures 1Be–g**).

### Induction of the *LIP5* Gene in *Arabidopsis* Leaves by ABA and Drought Stress

**FIGURE 2:**
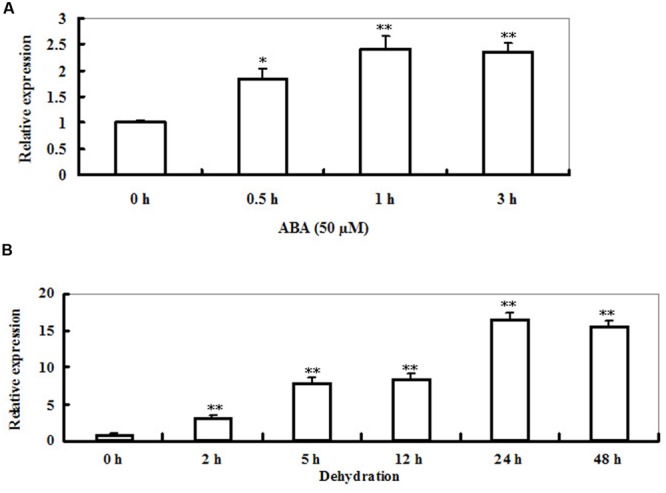
**Expression of *LIP5* in response to abscisic acid (ABA) or dehydration. (A)** ABA-induced *LIP5* expression revealed by qRT-PCR. Two-weeks-old wild-type seedlings were treated with 50 μM ABA for 0, 0.5, 1, and 3 h. *Actin2* was used as an internal control. **(B)** Dehydration-induced *LIP5* expression revealed by qRT-PCR. Two-weeks-old wild-type seedlings were placed on Whatman paper and then harvested at 0, 2, 5, 12, 24, and 48 h. In both **(A,B)** *Actin2* was used as an internal control. For each experiment, three technical replicates were conducted. Data shown are mean ± SE of three independent experiments. *T*-test, with ^∗∗^*P* < 0.01; *t*-test, with ^∗^*P* < 0.05.

Time-course analysis of *LIP5* transcript levels in *Arabidopsis* plants under ABA or dehydration stress was performed by qRT-PCR (**Figures 2A,B**). The transcript levels of *LIP5* were increased rapidly after 0.5 h, and then remained higher levels during 3 h period of ABA treatment with a peak at 1 h (about 1.5-fold increase in transcripts; **Figure 2A**). Accumulation of *LIP5* transcripts was first detected 2 h after the dehydration treatment and reached a maximal level at 24 h during 48 h period of the stress (**Figure 2B**). The *LIP5* transcripts were upregulated by 16-fold after 24 h of exposure to dehydration. These data suggest that *LIP5* could be involved in the responses to ABA or drought stress.

### Genetic Complementation of the *Arabidopsis lip5* Knockdown Mutants in Response to ABA

**FIGURE 3:**
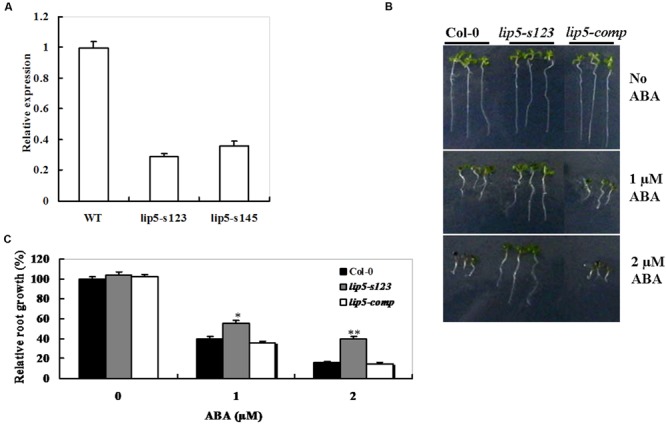
**Genetic complementation of the *lip5* mutant in response to ABA. (A)** Transcript levels of *AtLIP5* in the Col-0 and *lip5-s123* and *lip5-s145* mutants determined by RT-PCR. **(B)** Representative phenotypes of seedlings at 7 days after the end of stratification. Twenty-four hours after stratification, germinated seeds were transferred from ABA-free medium to medium containing 1 or 2 μM ABA. **(C)** Root growth measurements. Seedling root length was measured at 7 days after the end of stratification. Relative root growth compared with that on ABA-free medium is indicated. Data show the mean ± SE of three replicates. At least 30 seedlings per genotype were measured in each replicate. *T*-test, with ^∗∗^*P* < 0.01; *t*-test, with ^∗^*P* < 0.05. In both **(B,C)** experiments were repeated at least two times with similar results.

To explore the *LIP5* function in response to ABA treatment, two independent homozygous T-DNA insertion lines SALK_123717 (named *lip5-s123*) and SALK_145666 (named *lip5-s145*) were identified from the ABRC seed stock center. Analysis of the full-length cDNA and the genomic sequences revealed that *LIP5* is composed of six exons and five introns (**Supplementary Figure S1A**). The gene is located on chromosome IV of the *Arabidopsis* genome. In both lines, the T-DNA was inserted in the region of 300 bp upstream of the translational start site of *LIP5*. The T-DNA insertion positions are illustrated in **Supplementary Figure S1A** and homozygous mutants were verified using *LIP5* gene-specific and T-DNA border primers by PCR analysis (**Supplementary Figure S1B**). In both homozygous lines, a decrease of 60% approximately in abundance of *LIP5* mRNA was detected by qRT-PCR compared with the same aged wild-type plants (**Figure 3A**). Both T-DNA insertion lines had comparable levels in *LIP5* transcripts, so the *lip5-s123* line was chosen for further analysis.

The *lip5* mutant (*lip5-s123*), *lip5-s123/35S*-*LIP5* line (*lip5-comp*), along with wild-type (Col-0) were also assessed for their responses to ABA by investigating the retardation of seedling root growth. Under 1 or 2 μM of ABA, root growth of both wild-type and the *lip5-comp* plants was more severely inhibited than that of the *lip5* mutant (**Figures 3B,C**). Noticeably, the *lip5-comp* line is actually an over-expression line (*lip5-s123/35S*-*LIP5*), but it behaved like the wild-type plants in the ABA assays. To clarify these results, we detected the transcript levels of *LIP5* between wild type and the *lip5-comp* line used in the ABA assays using qRT-PCR. As a result, there were no significant differences in the transcript levels of *LIP5* between wild type and the *lip5-comp* line, which could explain the observation (Data not shown). These results demonstrated that *lip5* mutant was less sensitive to ABA than the wild type; moreover, *LIP5* effectively rescued the ABA-insensitive phenotypes of the *lip5* knockdown mutants.

### ABA Response of *LIP5* Mutant and OE Lines

**FIGURE 4:**
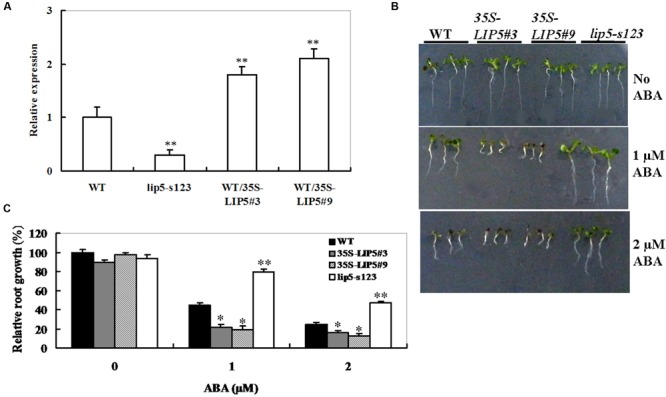
**Abscisic acid response of *lip5* mutants, overexpression lines and wild-type plants. (A)** Transcript levels of *LIP5* in the WT, *lip5* mutants and *WT/35S-LIP5* overexpression lines (named WT/35S-LIP5#3 and #9) determined by qRT-PCR. *T*-test, with ^∗∗^*P* < 0.01. **(B)** Representative phenotypes of seedlings grown on the medium containing ABA at 7 days after the end of stratification. Twenty-four hours after stratification, germinated seeds were transferred to medium containing 0, 1, or 2 μM ABA. **(C)** Root growth measurements. Seedling root length was measured at 7 days after the end of stratification. Relative root growth compared with that on ABA-free medium is indicated. Data show the mean ± SE of three replicates. At least 30 seedlings per genotype were measured in each replicate. *T*-test, with ^∗∗^*P* < 0.01; *t*-test, with ^∗^*P* < 0.05. In both **(B,C)** experiments were repeated at least two times with similar results.

To further investigate the function of *LIP5* in response to ABA, wild-type *Arabidopsis* plants overexpressing the *LIP5*, under the control of the 35S promoter, were generated. Five homozygous lines (T3 generation) were obtained, and two lines (*WT/35S-LIP5*#3 and #9) exhibiting high levels of transgene expression (**Figure 4A**) were selected for phenotypic characterization. The *LIP5* mutant (*lip5-s123*) and *LIP5* OE lines (*WT/35S-LIP5*#3 and #9) were also examined for their ABA response by investigating the retardation of seedling root growth. When grown on the medium with 1 or 2 μM of ABA, root growth inhibition of *lip5-s123* seedlings was less than that of the wild type, whereas both OE lines were more severely inhibited (**Figure 4B**). Relative root length determination also showed significant differences among the mutant, OE lines and wild-type plants under 1 or 2 μM of ABA (**Figure 4C**). These indicate that *lip5-s123* is less sensitive to ABA but OE lines are hypersensitive. Taken together, the contrasting ABA sensitivities between *lip5* mutant and *LIP5* OE lines suggest that *LIP5* may act as a positive regulator of ABA signaling during seedling development.

### *LIP5* Affects Drought Tolerance of Plants

**FIGURE 5:**
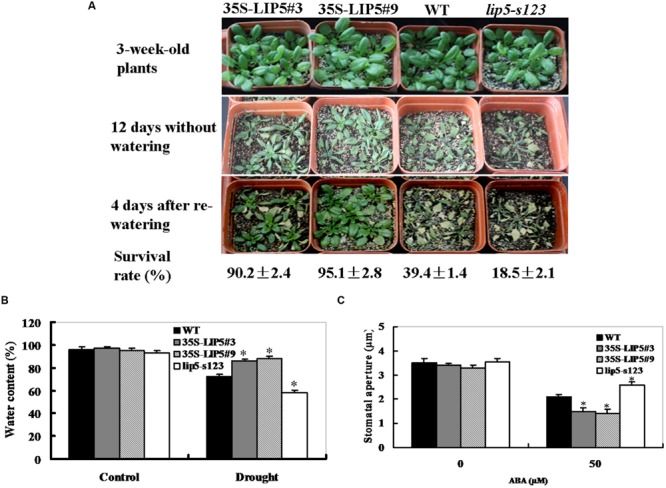
**Phenotypes of *lip5* mutants, overexpression lines and wild-type plants in response to drought stress. (A)** Drought tolerance of potted plants of wild-type, *lip5* mutant and overexpression *Arabidopsis*. Three-weeks-old *lip5-s123*, two *WT/35S-LIP5* lines (#3 and #9) and WT plants grown in soil in pots are subjected to drought stress by withholding water for 12 days. After re-watering for 4 days, percentage of plants survived was measured. Representative images were taken before drought (upper), at 12 days after drought treatment (middle) and at 4 days after re-watering (lower). **(B)** Water content. After 8 days without watering, leaf samples from transgenic, mutant and wild-type plants under stressed and control conditions were determined according to the procedure in Section “Materials and Methods.” Values are mean ± SE of three independent repetitive experiments from different plants. *T*-test, with ^∗^*P* < 0.05. **(C)** Measurement of stomatal aperture in response to ABA. Epidermal peels from 3-weeks-old different genotype plants were kept for 12 h in the dark, incubated under light in stomata-opening solution for 1.5 h, and then treated with 0 and 50 μM ABA for 3 h before stomatal apertures were measured under the microscope. Data show the mean ± SE of three replicates (*n* = 30). *T*-test, with ^∗^*P* < 0.05. In **(A–C)** experiments were repeated three times with similar results.

To test the role of *LIP5* in regulating drought response of plants, 3-weeks-old *35S:LIP5, lip5* mutant, and wild-type plants were used for drought tolerance assays in soil. As shown in **Figure 5A**, after 12 days without watering, most of the 35S:LIP5 plants survived the water stress and upper leaves of these plants were still green and fully expanded, whereas most of *lip5* and wild-type plants displayed severe wilting (especially for the *lip5* plants, all leaves were severely curled and partial leaves were turning yellow). After re-watering, 35S:LIP5 plants showed a high survival rate (more than 90%), whereas the corresponding survival rates were 39% for wild-type plants and 19% for *lip5* mutants (**Figure 5A**); indicating that *LIP5* plays a positive role in drought tolerance. Consistent with their drought-tolerant performance, the water content of both OE lines after 8 days without watering was significantly higher than that of the wild-type and *lip5* mutant plants (**Figure 5B**); suggesting that the improved drought tolerance of both OE lines resulted from decreased water loss from plants.

Several studies have established that high sensitivity to ABA leads to increased drought tolerance by stomatal aperture measurement (Lim and Lee, 2014). To determine whether the differential drought-tolerant phenotypes were associated with their enhanced ABA sensitivity, we measured ABA-mediated stomatal aperture (**Figure 5C**). In the absence of ABA, there was no obvious difference in stomatal aperture among wild-type, *lip5* mutant and OE plants. However, the stomatal pore sizes were significantly reduced in both OE plants compared with wild-type plants after ABA treatment. The stomatal apertures of wild-type and OE plants were reduced by 40 and 58–60%, respectively; whereas those of *lip5* plants were only reduced by 26% (**Figure 5C**); demonstrating that *LIP5* OE plants were more sensitive than the wild-type in ABA-induced stomatal closure, whereas the *lip5* mutant plants were less sensitive. These data indicate that *LIP5* may act in regulating ABA-mediated stomatal closure.

### Cell Endocytosis or Vesicle Trafficking Induced by Stress- or ABA in *LIP5* Mutant and Wild Type Plants

**FIGURE 6:**
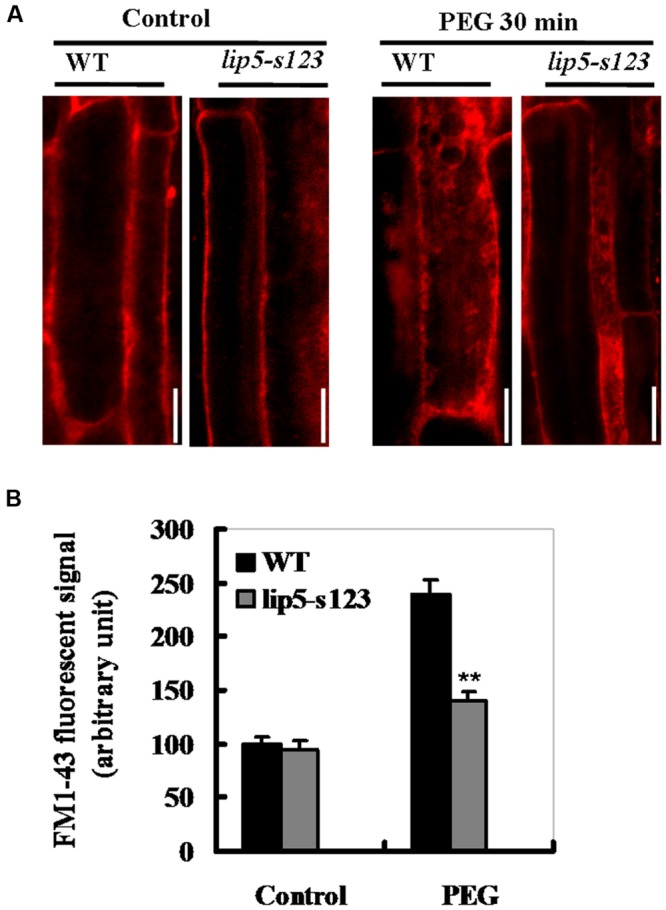
**Phenotypes of *LIP5* mutant and wild type plants in drought-induced endocytosis.** Ten days-old *lip5-s123* and WT seedlings on MS medium were carefully transferred to MS plates containing 0 or 10% of PEG 6000 for 30 min. Drought-induced endocytosis was analyzed using FM1-43 staining. **(A)** Representative confocal images of *Arabidopsis* root epidermal cells of WT and *lip5* mutant plants after FM1-43 staining. Bar = 50 μm. **(B)** Signal intensity of internalized FM1-43 in *Arabidopsis* root epidermal cells of WT and *lip5* mutant plants. Means and SE were calculated from images of 8 independent roots (10 images/root) for each genotype. *T*-test, with ^∗∗^*P* < 0.01. In both **(A,B)** experiments were repeated two times with similar results.

It has been recently shown that *LIP5* exerts its regulatory roles in pathogen- and salt-induced cellular vesicle trafficking (Wang et al., 2014, 2015). To examine whether different levels of tolerance between the mutant and wild type plants were also associated with vesicle trafficking under stress conditions, we compared WT and *lip5* mutant plants for drought-induced endocytic activities using FM1-43 as a fluorescent endocytosis marker. The membrane-selective FM1-43, whose fluorescent signals are located in a lipid-rich membrane, can enter the cells by endocytic vesicles derived from plasma membrane (Bolte et al., 2004). Without PEG (on MS medium), FM1–43 fluorescent signals were predominantly located at the plasma membrane as a result of the association of the dye with the lipid phase (**Figure 6A**, left lane). Moreover, no significant difference was observed in internalized FM1-43 signals between WT and *lip5* mutant root cells (**Figures 6A,B**). After 30 min-PEG (10%) treatment, the fluorescent signals at the plasma membrane were reduced and became diffusive and strong in the cytoplasm. This was clearly seen in the root epidermal cells of the wild type (**Figure 6A**, right lane). The intensity of internalized FM1-43 signals increased by almost 1.5 folds in WT roots (**Figure 6B**). Unlike WT plants, however, there was only 50% increase in the fluorescence signals in the *lip5* roots (**Figure 6B**). Also, ABA-induced vesicle trafficking in guard cells of leaf epidermis was observed. Without ABA treatment, the fluorescent signals were evenly associated with the plasma membrane in both wild-type and *lip5* guard cells (**Supplementary Figure S2A**). Moreover, there was no significant difference in internalized fluorescent signals between wild-type and *lip5* (**Supplementary Figure S2A**). Surprisingly, after 1 h-ABA treatment, the internalized fluorescent signals were increased at the plasma membrane and became intense. Moreover, the ABA-induced stomatal closure was clearer in the wild type than the mutant (**Supplementary Figure S2A**). The internalized FM1-43 signals increased by 180% in the wild type, but only 85% in the *lip5* (**Supplementary Figure S2B**). These results indicate that drought or ABA induces cell endocytosis or vesicle trafficking in a largely *LIP5*-dependent way.

### *LIP5* Affects Expression of Drought- or ABA-Responsive Genes

**FIGURE 7:**
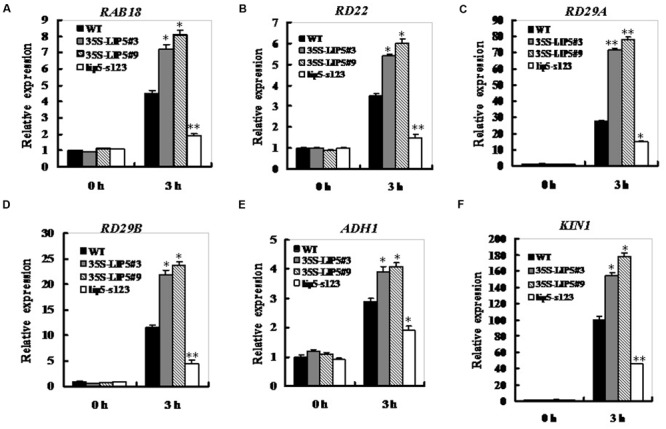
**Expression of ABA- and stress-responsive genes in the *lip5* and overexpression lines in response to ABA.** Three-weeks-old plants were treated with 50 μM ABA for 0 and 3 h, and expression of ABA- and stress-responsive genes *RAB18* **(A)**, *RD22* **(B)**, *RD29A* **(C)**, *RD29B* **(D)**, *ADH1* **(E)**, and *KIN1* **(F)** was assayed by qRT-PCR in seedlings of WT, *lip5*-s123, and two *WT/35S-LIP5* lines (#3 and #9). For each experiment, three technical replicates were conducted. Data shown are mean ± SE of three independent experiments. *T*-test, with ^∗∗^*P* < 0.01; *t*-test, with ^∗^*P* < 0.05.

Generally, drought sensitivity is correlated to transcripts levels of drought- or ABA-related genes (Xiong et al., 2002; Xia et al., 2013). The expression levels of *LIP5* are positively correlated with both ABA sensitivity and drought tolerance, so we next wondered whether LIP5 affected the expression profiles of ABA-induced or drought stress-related genes. Both LIP5 OE lines and *lip5* mutant together with wild-type seedlings were treated or mock treated with ABA to determine the expression of several drought- or ABA-inducible marker genes. qRT-PCR assays indicated that ABA treatment resulted in increased expression of several ABA-inducible marker genes, including *RAB18 (Responsive to ABA 18), RD22 (Responsive to dehydration 22), RD29A, RD29B, ADH1 (*alcohol dehydrogenase 1*)* and *KIN1* (Kurkela and Borg-Franck, 1992; Lang and Palva, 1992; Abe et al., 2003) in wild-type seedlings (**Figure 7**). The ABA-induced expression of these marker genes was generally intensified in both *LIP5* OE lines (increased averagely by 0.6∼1.7-fold at 3 h compared with the wild-type), but significantly attenuated in *lip5* seedlings (decreased by 0.4∼0.6-fold at 3 h compared with the wild-type; **Figures 7A–F**). These results indicate that the *LIP5* may function as a positive regulator in ABA-mediated drought tolerance by up-regulating expression of some ABA- or drought-responsive genes.

## Discussion

The MVB pathway plays essential roles in several eukaryotic cellular processes. Proper function of the MVB pathway requires reversible membrane association of the ESCRTs, a process catalyzed by SKD1 AAA ATPase. LIP5, a plant homolog of Vps twenty associated 1 (Vta1), is a positive regulator of MVB biogenesis (Haas et al., 2007). Moreover, LIP5 plays a critical role in pathogen/stress-responsive MVB biogenesis in plant stress responses (Wang et al., 2014, 2015). However, little is known about the relationship between the ABA signaling and LIP5-mediated stress responses. We report here *LIP5* plays a positive role in regulating ABA signaling and drought response. Overexpression of *LIP5* leads to ABA-associated phenotypes such as ABA hypersensitivity in seedling growth (**Figures 4B,C**), enhanced stomatal closure (**Figure 5C**), reduced water loss (**Figure 5B**), and, therefore, increased drought tolerance (**Figure 5A**). On the contrary, the *lip5* mutant showed ABA-insensitive phenotypes and reduced drought tolerance (**Figures 3–5**). These data indicate that *LIP5* may be a positive regulator in the ABA signal transduction pathway.

We considered the possibility that *LIP5* positively participated in ABA signaling regulation in response to drought stress in *Arabidopsis* based on the following reasons. First, *LIP5* was rapidly induced by ABA and water stress (**Figures 2A,B**). Second, the *lip5* knockdown mutants and *LIP5*-overexpressing transgenic plants exhibited opposite sensitivities to ABA in seedling growth (**Figures 4B,C**). Third, *LIP5* positively regulated ABA-induced stomatal closure, which may reduce transpiration water loss in response to drought stress (**Figures 5B,C**). Phenotypic analysis indeed demonstrated that *LIP5*-overexpressing transgenic plants were highly tolerant to drought stress, as they survived without watering for 12 days (**Figure 5A**). In contrast, the *lip5* mutant line was susceptible to the stress and lost leaf water faster than wild-type plants (**Figures 5A,B**). Finally, *in silico* analysis of *LIP5* promoter region (1, 281-bp upstream of *LIP5* ORF) with the PlantCARE program (Lescot et al., 2002) identified several consensus *cis*-acting elements related to stress responses such as ABRE (ABA-responsive element), TC-rich repeats and TCA-element (Supplementary Table S2). These *cis*-acting elements are involved in responses to ABA, drought or stress defense in *Arabidopsis* (Nakashima et al., 2006). Noticeably, both T-DNA insertion mutants, in which the T-DNA was inserted into the region close to the second ABRE motif in the promoter of the *LIP5* gene, showed reduced ABA sensitivity in our study. Overall, these results led us to propose that LIP5 may be a positive regulator of an ABA-dependent response to drought stress in *Arabidopsis*.

LIP5 is a positive regulator, but it is not an essential component of MVB biogenesis. Wang et al. (2014, 2015) demonstrated that the hypersensitivity of *lip5* knockout mutants to pathogen infection, heat or salt stress is associated with defects in stress-induced formation of endocytic vesicles and intracellular MVBs. This suggests LIP5 has a critical role of pathogen/stress responsive MVB biogenesis in broad plant stress responses. However, there has been no direct genetic evidence for the role of MVBs in affecting ABA signaling as mutations of genes essential for MVB biogenesis are often lethal (Haas et al., 2007; Spitzer et al., 2009). In this study, the *lip5* mutants decreased sensitivity to ABA and reduced drought tolerance (**Figures 3–5**). Using the FM1–43 staining, we further observed that stress- or ABA-induced endocytic activities were increased in wild-type plants but not in the *lip5* mutant plants (**Figure 6**, Supplementary Figure S2). This suggested that LIP5 is necessary for stress-induced vesicle trafficking. We further demonstrated that *LIP5* over-expression plants were more sensitive than the wild-type in ABA-induced stomatal closure, whereas the *lip5* mutant plants were less sensitive (**Figure 5**); indicating that *LIP5* may act in regulating ABA-mediated stomatal closure. Noticeably, it has been reported that ABA triggers endocytosis of K^+^ channel proteins at the plasma membrane in plant guard cells to control ion transport and transpiration under water stress (Sutter et al., 2007). Based on this report and our results, we speculate that *LIP5-*regulated endocytosis might be involved in ABA-induced stomatal closure under drought conditions.

In correspondence with these ABA- or drought tolerance-related phenotypes, expression of several drought- or ABA-responsive marker genes, including *RAB18, RD22, RD29A, RD29B, ADH1*, and *KIN1* was significantly down-regulated upon ABA exposure in the *lip5* mutant seedlings (**Figure 7**). *RD29A* is a drought- and ABA-inducible gene and its expression in the *lip5* mutant could be due to the reduction of the upstream bZIP family transcription factors (Shinozaki and Yamaguchi-Shinozaki, 1997). However, it is not sure which of the bZIP factors affected *RD29A* expression. The expression of *RAB18* was significantly reduced in the *lip5* mutant, similar to that of *RD22* (**Figures 7A,B**). Several MYC and MYB binding elements have been found in the promoter regions of *RAB18* and *RD22*, indicating that both genes may be regulated by the two classes of transcription factors MYB and MYC (Zhang et al., 2007). In the *lip5* mutant, reduced expression of *LIP5* attenuated ABA signal transduction, which might result in decreased expression of MYC and MYB and finally affect the expression of *RAB18* and *RD22*. Based on previous and our results, it is reasonable to speculate that under drought conditions, *LIP5* promotes cell endocytosis and vesicle trafficking, which might accelerate trafficking and sorting of some regulatory proteins such as bZIP, MYC or MYB, and finally results in up-regulation of some ABA-induced downstream genes (such as *RAB18, RD22, RD29A*, et al.) and ABA response. Future work will be needed to dissect the mechanisms in detail by which the LIP5-regulated MVB biogenesis is involved in ABA signal transduction using the isolated *lip5* mutants and *LIP5*-overexpression lines.

## Author Contributions

Zongliang Xia designed the research. YH, YW, QC, Ziwei Xu, WZ, and Zongliang Xia performed research and conducted data analyses. Zongliang Xia wrote the manuscript.

## Conflict of Interest Statement

The authors declare that the research was conducted in the absence of any commercial or financial relationships that could be construed as a potential conflict of interest.

## Footnotes

1 http://bioinformatics.psb.ugent.be/webtools/plantcare/html/

## Supplementary Material

FIGURE S1 **Molecular identification of *Arabidopsis lip5* mutants. (A)** Structure of the *LIP5* locus with the T-DNA insertion site in *lip5-s123* and *lip5-s145* mutants. The insertion site is marked by a white arrow, exons are indicated as white boxes, and untranslated regions by black boxes. P1, forward primer; P2, reverse primer; LBb1, primer specific to the T-DNA left border. **(B)** Diagnostic PCR of the T-DNA inserted in two different loci of *LIP5*. DNA from homozygous insertion lines of *lip5-s123* and *lip5-s145* were used. M, molecular mass markers. Primers used for PCR are indicated above each lane.

FIGURE S2 **Phenotypes of *LIP5* mutant and wild type plants in ABA-induced endocytosis in leaves.** Two weeks-old *lip5-s123* and WT plants were treated by spraying 50 μM ABA, and after 1 h, ABA-induced endocytosis was analyzed from control and treated *Arabidopsis* leaf epidermis using FM1-43 staining. **(A)** Representative confocal images of *Arabidopsis* guard cells of WT and *lip5* mutant plants after FM1-43 staining. Bar = 10 μm. **(B)** Signal intensity of internalized FM1-43 in *Arabidopsis* leaf guard cells of WT and *lip5* mutant plants. Means and SE were calculated from images of 10 independent leaves for each genotype. *T*-test, with ^∗∗^*P* < 0.01. In both **(A,B)** experiments were repeated two times with similar results.
