# Supplementary material for: The Arabidopsis LYST INTERACTING PROTEIN 5 Acts in Regulating Abscisic Acid Signaling and Drought Response
Source: Front Plant Sci. 2016 Jun 1;7:758. doi: 10.3389/fpls.2016.00758 (PMC4887465; doi:10.3389/fpls.2016.00758)
Supplement: Supplementary file 1 [file Table_1.DOC]

**Table S1 PCR primers used in this study**.

Note: Basic residues are indicated in bold.

| Primer name | Primer sequence^*^ | Use |
| --- | --- | --- |
| P1  P2  LBb1  LIP5-QF  LIP5-QR  RD22-QF  RD22-QR  RD29A-QF  RD29A-QR  RD29B-QF  RD29B-QR  KIN1-QF  KIN1-QR  RAB18-QF  RAB18-QR  ADH1-QF  ADH1-QR  LIP5-F  LIP5-R  LIP5P-F  LIP5P-R  Actin2-F  Actin2-R | 5'-GATGGAGTTCCTTTACTCGCC-3'  5'-TCTTAGTTCTCTCGCTCTGCG-3'  5'-ATTTTGCCGATTTCGGAAC-3'  5'-GCTGCGAGCATCTTCTTTGAAATTC-3'  5'-TATCATCATCCACAGGATCACCTGG-3'  5'-ATGGCGATTCGTCTTCCTCTGATC-3'  5'-ACTCCGCCTTTACCTACTTGGACG-3'  5'-CAGAGGAACCACCACTCAACACA-3'  5'-CTCTAGGTTTACCTGTTACGCCTG-3'  5'-ATGGAGTCACAGTTGACACGTCCT-3'  5'-CTTCTGGGTCTTGCTCGTCATACT-3'  5'-ATGTCAGAGACCAACAAGAATGCC-3'  5'-CTACTTGTTCAGGCCGGTCTTG-3'  5'-ATGGCGTCTTACCAGAACCGTCCA-3'  5'-ACCACCACTTTCCTTGTGGAGTTG-3'  5'-ATGTCTACCACCGGACAGATT-3'  5'-CGAGTGGCAATGACGACACTC-3'  5′-AGGGATCCATGTCGAACCCAAACGAAC-3′  5′-CATCTAGATCAGTGACCGGCACCGGC-3′  5′-AGGAATTCTGCTGGGAATACTGAACCT-3′  5′-CCAAGCTTTCGCAGAAAGGACCTCTAA-3′  5'-TTGTGCTGGATT CTGGTGATGG-3'  5'-CCGCTCTGCTGTTGTGGTG-3' | Verification of homozygous *lip5* T-DNA SALK lines  qRT-PCR for *LIP5* expression  qRT-PCR for *RD22* expression  qRT-PCR for *RD29A* expression  qRT-PCR for *RD29B* expression  qRT-PCR for *KIN1* expression  qRT-PCR for *RAB18* expression  qRT-PCR for *ADH1* expression  Construction of *LIP5* over-expression vector  Construction of *LIP5 promoter-GUS* fusion expression vector  qRT-PCR for *Actin2* expression |

^*^The underlined nucleotides constitute *Bam*HI (GGATCC), *Eco*RI (GAATTC), *Hin*dIII (AAGCTT), or *Xba*I (TCTAGA) restriction enzyme digestion sites.
