# Supplementary material for: The Arabidopsis LYST INTERACTING PROTEIN 5 Acts in Regulating Abscisic Acid Signaling and Drought Response
Source: Front Plant Sci. 2016 Jun 1;7:758. doi: 10.3389/fpls.2016.00758 (PMC4887465; doi:10.3389/fpls.2016.00758)
Supplement: Supplementary file 2 [file Table_2.DOC]

**Table S2 Stress-related cis-acting regulatory elements identified in the promoter region of *LIP5***.

| **Site name** | **Position** | **Strand** | **Sequence** | **Function** |
| --- | --- | --- | --- | --- |
| ABRE | 98 | + | GCAACGTGTC | cis-acting element involved in the abscisic acid responsiveness |
|  | 441 | **–** | CACGTG | cis-acting element involved in the abscisic acid responsiveness |
| TC-rich repeats | 396 | + | ATTTTCTTCA | cis-acting element involved in stress and defense responsiveness |
| MBS | 1133 | + | CGGTCA | MYB binding site |
| HSE | 1236 | + | AAAAAATTTC | cis-acting element involved in heat stress responsiveness |
| TCA-element | 253 | + | TCAGAAGAGG | cis-acting element involved in salicylic acid responsiveness |
| ARE | 321 | + | TGGTTT | cis-acting regulatory element essential for the anaerobic induction |
