# Supplementary figures and images for: The Arabidopsis LYST INTERACTING PROTEIN 5 Acts in Regulating Abscisic Acid Signaling and Drought Response
Source: Front Plant Sci. 2016 Jun 1;7:758. doi: 10.3389/fpls.2016.00758 (PMC4887465; doi:10.3389/fpls.2016.00758)

# Suppl Fig.1

**A**

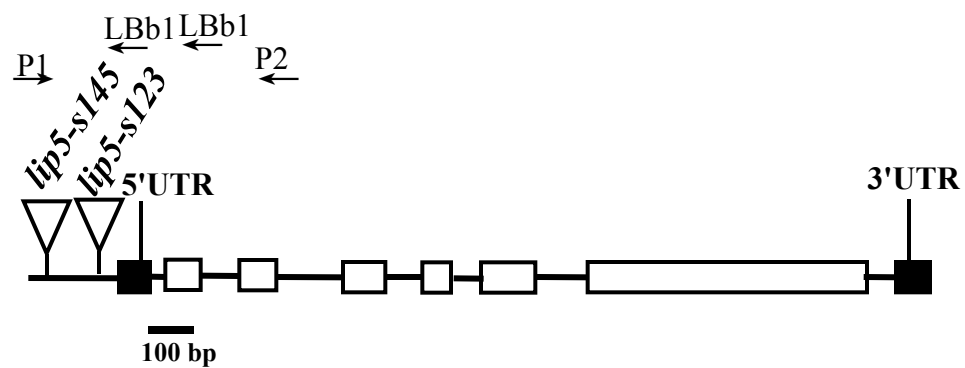

**B**

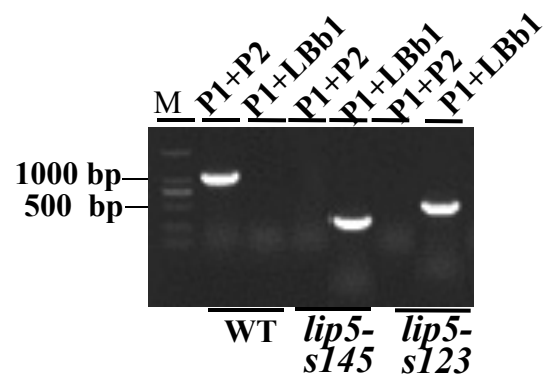

Supplement: FIGURE S1 — Molecular identification of Arabidopsis lip5 mutants. (A) Structure of the LIP5 locus with the T-DNA insertion site in lip5-s123 and lip5-s145 mutants. The insertion site is marked by a white arrow, exons are indicated as white boxes, and untranslated regions by black boxes. P1, forward primer; P2, reverse primer; LBb1, primer specific to the T-DNA left border. (B) Diagnostic PCR of the T-DNA inserted in two different loci of LIP5. DNA from homozygous insertion lines of lip5-s123 and lip5-s145 were used. M, molecular mass markers. Primers used for PCR are indicated above each lane. [file Image_1.PDF]

Suppl Fig.2

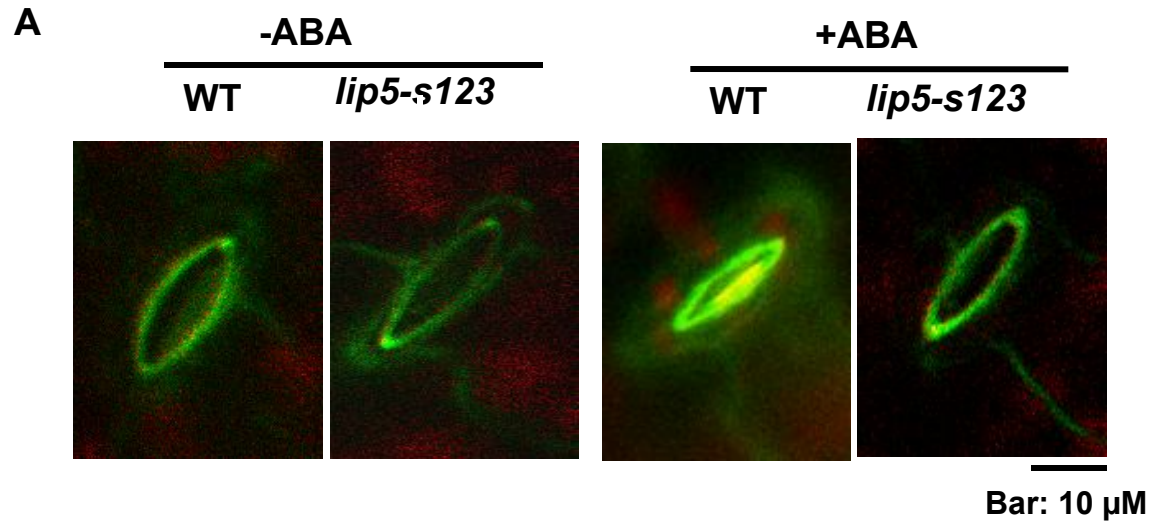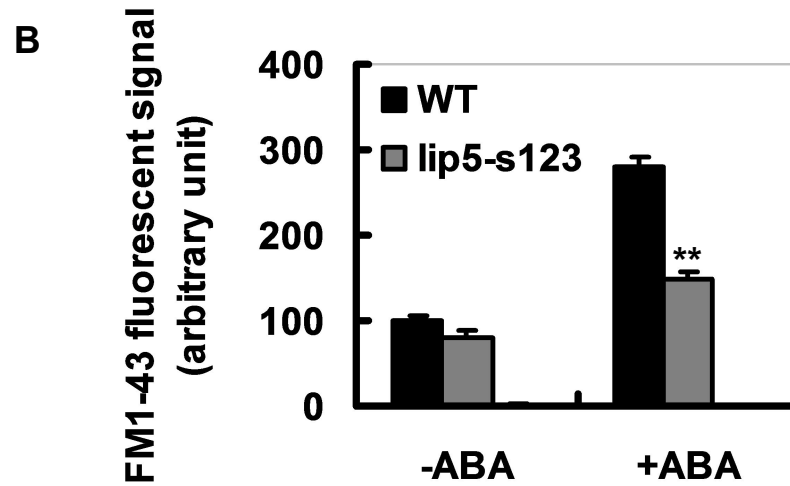

Supplement: FIGURE S2 — Phenotypes of LIP5 mutant and wild type plants in ABA-induced endocytosis in leaves. Two weeks-old lip5-s123 and WT plants were treated by spraying 50 μM ABA, and after 1 h, ABA-induced endocytosis was analyzed from control and treated Arabidopsis leaf epidermis using FM1-43 staining. (A) Representative confocal images of Arabidopsis guard cells of WT and lip5 mutant plants after FM1-43 staining. Bar = 10 μm. (B) Signal intensity of internalized FM1-43 in Arabidopsis leaf guard cells of WT and lip5 mutant plants. Means and SE were calculated from images of 10 independent leaves for each genotype. T-test, with ∗∗P < 0.01. In both (A,B) experiments were repeated two times with similar results. [file Image_2.PDF]
